# Preparation of Super Absorbent and Highly Active Fish Collagen Sponge and its Hemostatic Effect *in vivo* and *in vitro*

**DOI:** 10.3389/fbioe.2022.862532

**Published:** 2022-03-10

**Authors:** Lei Wang, Wenjun Li, Yan Qu, Kai Wang, Kangning Lv, Xiaoli He, Song Qin

**Affiliations:** ^1^ Key Laboratory of Biology and Bioresource Utilization, Yantai Institute of Coastal Zone Research, Chinese Academy of Sciences, Yantai, China; ^2^ University of Chinese Academy of Sciences, Beijing, China; ^3^ Department of Dermatology, The Affiliated Hospital of Weifang Medical University, Weifang, China; ^4^ Department of Orthopedics, The Affiliated Yantai Yuhuangding Hospital of Qingdao University, Weifang, China; ^5^ Yantai University, Yantai, China

**Keywords:** hemostatic material, high porosity, high blood absorption rate, fish collagen, adhesion

## Abstract

Effective control of acute wound hemorrhage caused by battlefields, car accidents, natural disasters can highly improve patients’ survival rates. Nevertheless, hemostatic materials on present market have various defects and limitations. This study utilizes tilapia to extract macromolecular type I collagen to prepare a new hemostatic sponge for controlling acute wound bleeding. The extracted fish collagen has high purity, uniform molecular size and high hydroxyproline content. The peptide chain structure and natural high-level structure are intactly preserved. The infrared absorption spectrum showcases that it preserves all the characteristic absorption bands of type I collagen. The developed hemostatic sponge has a uniform honeycomb-shaped porous structure and high water absorption capacity. The biological safety test illustrates that the sponge cell has good compatibility and it will not trigger any inflammatory reaction or immune rejection reactions in the body. The sponge cell could be degraded gradually and completely, which has good biocompatibility and degradation performance. The result of *in vitro* experiments *shows* that certain groups or structures in fish collagen molecules can combine specific sites on the surface of blood cells and platelets, which can quickly activate platelets and coagulation system to obtain better coagulation function. The result of *In vivo* experiments further shows that the fish collagen sponge has fast coagulation speed and low bleeding during the hemostasis process of rabbit ear arteries and rat liver wounds, which proves that it has excellent coagulation performance.

## Introduction

Blood is the most important fluid connective tissue in the human body. In the human body, blood plays a variety of physiological functions such as gas transportation, material transportation, immune detection and etc., which is an important component to maintain normal operation within the body (Hoffman et al., 2004; Li et al., 2017; Makhov and Zakharov, 2018; Wang et al., 2019). Hemorrhage cause by wars, car accidents and trauma around the world causes mortality of many soldiers and civilians every year (Eastridge et al., 2012; Shlaifer et al., 2017). Bleeding is the main reason for death amongst trauma patients. Effective and rapid control of bleeding can save precious time for following-up treatment and therefore reduce mortality effectively. (Wang et al., 2018). There are various types of rapid hemostasis methods in this regard, and rapid hemostatic materials become indispensable products for clinical and emergency treatments.

At present, there are various hemostatic products and materials in clinical applications, which can be classified as non-absorbable and absorbable hemostatic materials. Applying non-absorbable hemostatic material is likely to cause wound infection, which limits its applications in clinical, battlefield and emergency care (Shi-fu et al., 2014). On the contrary, absorbable hemostatic materials are widely studied and investigated. The absorbable hemostatic materials that are currently utilized mainly include chitosan, sodium alginate, hemostatic gelatin, carboxymethyl cellulose, hemostatic sponge, oxidized regenerated cellulose, zeolite hemostatic materials, peptides (Zheng et al., 2021) and etc. However, due to the high rate of rebleeding after hemostasis, together with other issues such as the small area of hemostasis, the embolism risk, the induction of immune rejection or allergic reactions, applications of existing hemostatic products are very limited (Kozen et al., 2008; Kunio et al., 2013; Liang et al., 2019). The questions including how to achieve a high bloodsucking rate with good mechanical properties, how to maintain a stable structure with good biocompatibility, and how to obtain simple operation with effective hemostasis are the current research hotspots (Wang et al., 2021). Meanwhile, we need to avoid secondary infection, tissue damage, and induction of immune rejection to generate hemostatic material with good biological safety.

Collagen is widely utilized in the field of tissue engineering as it has accomplished good application effects in soft as well as hard tissue repairing (Pawelec et al., 2016; Preethi Soundarya et al., 2018). Studies have demonstrated that collagen has good hemostatic effects under natural conditions, which is an ideal wound hemostatic material. After contacting with blood on the wound surface, the groups on the collagen could match the protein signal receiver on the phospholipid bilayer recognition channel of the cell surface regarding the wound surface, which triggers the cells to secrete coagulation factors and stimulate platelets to produce coagulation factors. The original coagulation pathway eventually leads to platelet formation and limiting blood loss effectively. The complete platelet aggregation cascade can exclusively be induced by collagen (Muthukumar et al., 2014; Mahesh et al., 2015; Six et al., 2017). Monomer and fibrillar collagen effectively support platelet adhesion, while the natural triple helix structure of collagen and the polymerization of monomer collagen are necessary to induce platelet aggregation and secretion. Collagen shows fast integrin-mediated adhesion, while denaturing collagen does not. This illustrates the importance to keep the peptide chain of collagen intact and clarifies that collagen should have intact high-level structure, naturalness, and active binding sites during the manufacturing process (Shekhter et al., 2019). At present, there are many types of collagen hemostatic sponges in clinical applications, mostly are derived from terrestrial animals such as cattle or pigs. Currently, because of animal ethics, religious beliefs, and zoonotic diseases, their applications are quite limited.

Studies have validated that aquatic animals’ collagen is similar to that of terrestrial mammals in amino acid structure. Glycine accounts for about 30% of the total amino acid contents, and the percentage for proline hydroxylation is 35–48%. Methionine, isoleucine, and tyrosine acid contents are slightly lower than that of terrestrial mammals. The RGD amino acid sequence composed of arginine (Arg)-glycine (Gly)-aspartic acid (Asp) is the best representative of the sequence with cell adhesion. Aquatic animal collagen has a similar amino acid sequence structure (Silva et al., 2014). Therefore, it is convenient for cells to adhere to the spatial structure and guides tissue regeneration. It is an ideal raw material for medical usage. In addition, the large number of diaminodicarboxylic groups exist in the peptide chain makes aquatic animals’ collagen extremely hydrophilic and hemostatic (Chen et al., 2019). It has a similar hemostatic mechanism to terrestrial animal-derived collagen, which can be employed as alternative raw materials for similar hemostatic materials.

Aquatic animal collagens specifically type I and V are mainly derived from fish. At present, fish skin and scales and other tissues contained with high collagen content are mainly utilized for material extraction. Because the tissues contain cells, lipids, nucleic acids (Lim et al., 2019), etc., the extraction principle is to remove impurities and immunogenicity and retain collagen integrity at the same time. Compared with land animals, tilapia have a wide range of cultivation, strong adaptability, and wide sources. The structure of fish skin is simple and contains high content of type I collagen, so the extraction process is relatively simple, easy to remove impurities, immunogenic substances, etc. The extracted type I collagen has the characteristics of high hydrophilic, and has higher thermal stability compared with type I collagen of cold-water fish. The purpose of this investigation is to improve the existing extraction process and method, which can effectively shorten the extraction time, minimize damage and loss, obtain high-quality and complete structure of natural macromolecular fish collagen, and prepare collagen according to the requirements of hemostatic materials. In this manner, hemostatic sponge characterize by high porosity, large contact area, high water absorption and good hemostatic properties would be generated. We aim to effectively cut off the telopeptide structure of fish collagen and remove its immunogenicity by utilizing enzymatic hydrolysis technology. We use multi-stage membrane filtration technology to replace traditional methods for salting out and dialysis, which achieves impurity removal and bacteria filtering, separation and purification. The liquid concentration is completed and it helps reduce extract time. Present researches use physicochemical and molecular biology, cell biology, and pathology methods to test the physicochemical and biological properties of fish collagen hemostatic sponge. We compare fish collagen hemostatic sponge with bovine collagen hemostatic sponge and oxidized regenerated fiber *in vivo* and *in vitro*, and find differences among them in aspects of hemostatic performance and hemostatic mechanism.

## Materials and Methods

### Materials

Fish collagen hemostatic sponges are prepared by our laboratory utilizing tilapia skin. Both bovine collagen and oxidized regenerated cellulose are purchased from conventional medical device manufacturers. Hydroxyproline detection kit. Rat anticoagulated whole blood (freshly collected by SD rats during the experiment). All animal experiments are approved by the Animal Ethics Committee in Yantai Langdi Biotechnology Co., Ltd.

### Methods

#### Collagen Extraction and Sponge Preparation

**FIGURE 1:**
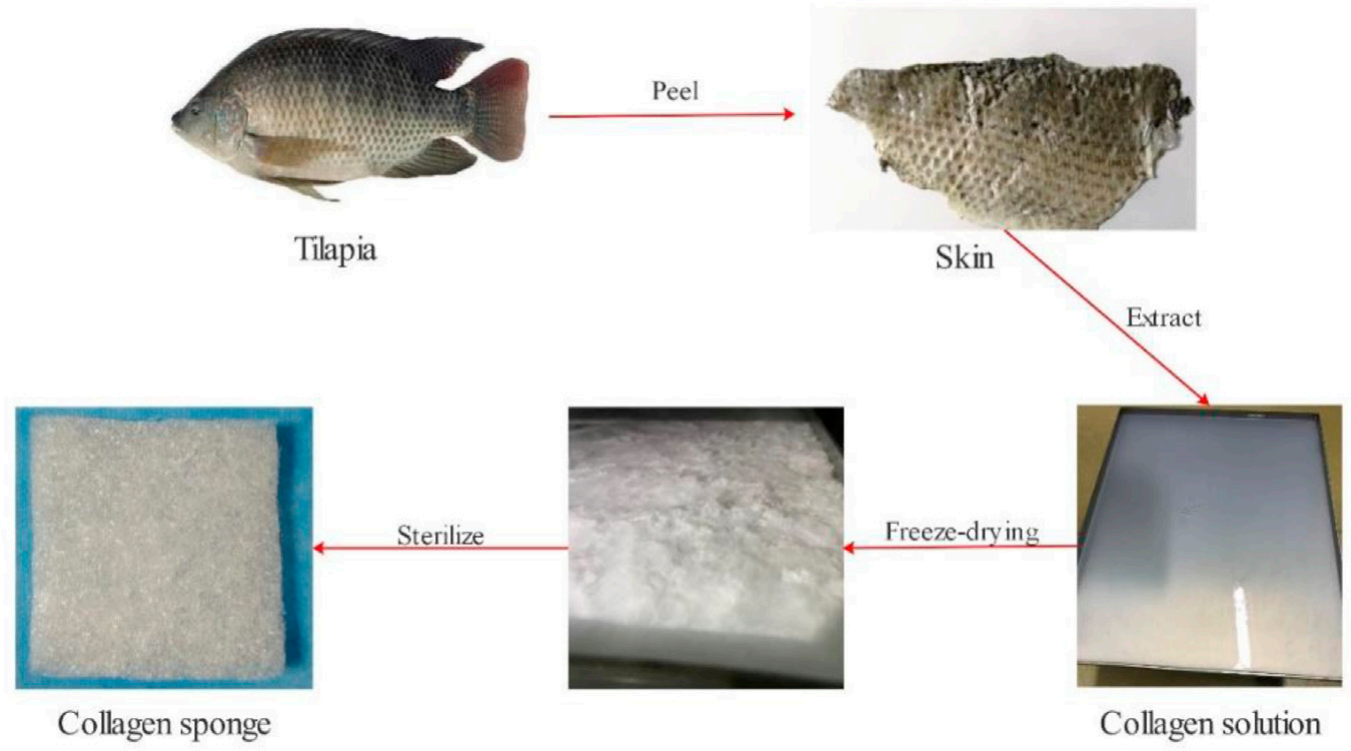
Extraction of tilapia collagen protein and preparation of sponge.

Our team employs low-temperature enzymatic hydrolysis and hierarchical membrane filtration technology for fish collagen extraction (Figure 1). We take 50 g tilapia fish skin, cut it into 0.5 cm*0.5 cm slices, and rinse with pure water for 3 times. We soak them in 1.3 L of 8% NaCl solution for 4 h and filter out the solution (remove impurities such as oil from fish skin). Then, we use 1.5 L of 0.6% SDS solution to soak for 3 h and filter out the solution (remove impurities such as oil from fish skin). After washing them with pure water for 3 times, we add 5% n-butanol solution (remove fat). We stir them at 60 rpm for 12 h and filter out the solution. Afterwards, we add 2 L of 0.2M NaOH solution into the reactor and stir with the same speed for 12h, and we filter out the solution (remove miscellaneous proteins). We add 1M acetic acid solution and stir for 48 h. We add pepsin and stir for 48 h. We transfer the reaction liquid to a centrifuge at 15000 rpm for 20 min. Our team let the solution pass through the large size pore to remove sediments. The small-pore filter membrane and the multi-stage ultrafiltration membrane are employed to remove large particles, microorganisms, small molecular substances and salt under the 0.45 Mpa input pressure, and finally we obtain a sterile collagen concentrate with a molecular weight of ≥100 KDa. We put the liquid in a freeze dryer for 72 h to obtain fish collagen spongesand sterilize it with Co60 before usage.

### Physical and Chemical Properties

We mark1g fish collagen sponge as M0 and put it in 100 ml pure water and soak for 3 days at room temperature until it is fully swelled. After water absorption, its weight achieves M1. We calculate the water absorption rate by the formula: *water absorption rate* (%) = (M1-M0)/M0, repeating 10 times. We cut the collagen sponge into 0.3 cm*0.3 cm cubes. After spraying gold, we observe the surface, porosity, pore size and internal structure of the sponge through electron microscope. We use 100 ml pure water to configure 1 mg/ml collagen solution, and carry through SDS-PAGE electrophoresis. We observe the molecular weight of collagen and utilize a hydroxyproline kit to measure its hydroxyproline content. Finally, Fourier infrared spectrometer is employed to analyze the collagen to observe and judge its characteristic absorption band.

### Cytotoxicity

We take 0.5 g fish collagen sponge and put it into a sterile Erlenmeyer flask. Our team add 300 ml RPMI-1640 to it and put it in a constant temperature shaker at 37°C with 50 rpm for 12 h and obtain the extract finally. L-929 mouse fibroblasts are cultured with the RPMI-1640 system containing 10% FBS, and the cells in the exponential growth phase of 48–72 h after passage are inoculated into new cell culture flasks. The inoculation cell concentration is 4*10^4^/ml. After 24-h culturing process, the original culture solution is removed. 15 ml of RPMI-1640 culture solution is added to the control group, while 15 ml of culture solution containing 50% extract is added to the experimental group. After being cultured to the second, fourth, and seventh day, three parallel culture flasks are taken from each group and the cell morphology is observed and counted. Cell Proliferation Inhibition Index (CPII) is determined by 100% - (average cell density of the experimental group/the average cell density of the control group × 100%), from which we judge the collagen sponge cytotoxicity.

Live/dead staining: Collagen sponge and L929 cells (cell concentration 4*10^4^/ml) were placed into a 48-well plate at the same time. After culturing for 1, 3 and 7 days, we replace the normal cell culture medium with 1.5 μL propidium iodide and 1 μL calcein-AM (Calcein-AM/PI Double Staining Kit, Japan) in PBS solution, and incubate for another 30 min. After the sample was gently washed with PBS, it was observed under a fluorescence microscope.

### Hemolysis Rate

We take 2 ml fresh anticoagulant blood from healthy SD rats and add 8 ml normal saline to dilute it. We take 5 g of collagen sponge from the experimental group and put it into a test tube and add 10 ml normal saline. For the negative control group, we add 10 ml normal saline to the test tube. For the positive control group, we add 10 ml distilled water to the test tube. We then add 0.2 ml SD rats blood to test tubes from three groups, which are mixed thoroughly and incubated at 37°C for 60 min. We take out the liquid in each tube and centrifuge them at 2500 rpm for 5 min. We then take the supernatant and measure the absorbance with an ultraviolet spectrophotometer at 545 nm wavelength, and repeat this process for three times to take the average. Hemolysis rate is defined as (sample absorbance-negative control absorbance)/(positive control absorbance-negative control absorbance) ×100%, repeating 10 times.

### Skin Sensitization Test

We used seven healthy female SD rats and remove their back hair. We apply the fish collagen solution (10 mg/ml) on their left spine (1 cm*1 cm), and apply the excipient on their right spine (1 cm*1 cm). After 12 h, we clean the coated area and observe local skin reactions including erythema, edema and its extent at 0, 4, 24, 48, and 72 h respectively.

### Degradation *in vivo*

28 SD rats are randomly divided into four groups and each group consists of seven rats. After the rats are anesthetized with 10% chloral hydrate at a dose of 0.4ml/100g, their backs are depilated within the 1 cm*1 cm area. After alcohol disinfection, we utilize a scalpel to cut a 0.5 cm full-thickness wound on the skin and embed 0.1 g sterilized collagen sponge under the skin with tweezers, suture the skin, and later, observe the rats’ physical and psychological performances. On the 3rd, 7th, 14th and 28th day after the operation, one group of rats are sacrificed respectively, and the embedded fish collagen sponge is taken out, which is fixed with 4% paraformaldehyde. We make routine paraffin section, HE and Masson staining to observe the wrapped and degraded materials with inflammatory cell infiltration.

### Bloodsucking Rate

0.1 g fish collagen, 0.1 g bovine collagen, and 0.1 g oxidized regenerated cellulose (M0) are soaked completely in rats’ anticoagulated whole blood. After the blood is fully absorbed, our team employs an electronic balance to weigh the three materials (M1). Each sample is tested three times under the same conditions. We calculate the blood absorption rate (A) of the three materials based on the weight differences before and after the bloodsucking. We compute the bloodsucking rate (A) by the formula: (M1-M0)/M0×100% and repeat 10 times.

### Blood Clotting Index (BCI)

With taking bovine collagen sponge and oxidized regenerated cellulose as controls, we use coagulation index (BCI) to evaluate the coagulation performance of fish collagen sponge *in vitro*. We cut the three materials into 0.5 × 0.5 × 0.5 cm^3^ cubes respectively and put them into clean beakers. We put one piece of material in each beaker, transfer all the beakers with the materials into a water bath, and leave it at 37°C for 5 min. Then, we drop 0.1 ml anticoagulated whole blood on each piece of material and immediately add 200 ul 0.2M calcium chloride solution. Five minutes later, we add 25 ml deionized water to each beaker and transfer itto a shaking water bath in the pot at 37°C and 50r/ min for 5 min. After this operation we take out the solution from the beaker and use an ultraviolet-visible spectrophotometer to measure the absorbance value (A) of the solution in each beaker at 545 nm. We take 0.1 ml blood and add 25 ml deionized water to measure the absorbance value (A) at the same wavelength as the reference value. We repeat this process five times to calculate the coagulation index BCI by using the formula BCI = 100×A sample/A reference.

### Whole Blood Clotting Time

We use bovine collagen sponge and oxidized regenerated cellulose as controls to measure the coagulation time of whole blood *in vitro*. We take a 10 ml test tube and add 0.2 g sample to it, and repeat this process for each material. We put the material in a water bath at 37°C for 10 min. We take 3 ml rat anticoagulated whole blood and add 0.2M CaCl_2_ solution to the test tube for recalcification. We repeat the same operation for all tubes. We put the test tube containing recalcified whole blood into a 37°C water bath and take out the first test tube of each material every 10 s, and observe whether the blood is flowing at an angle. The other test tubes are maintained in the water bath. When the blood in the first test tube is tilted and does not flow, we use the same way to observe the blood status in the second test tube. Until the blood in the third test tube is completely coagulated, we stop the timing and record the overall coagulation time. Once the coagulation time exceeds 30 min, the material will be recorded as non-coagulated and the same operation will be repeated 5 times.

### Dynamic Coagulation Test

We cut fish collagen sponge, bovine collagen sponge, and oxidized regenerated cellulose into round pieces with a diameter of 1.5 cm and thickness of 0.5 cm. Seven of each material are placed in a disposable clean petri dish. We set seven time points and the duration between every two neighboring points is 5 min. We take 200 ul fresh rat whole blood and drip it onto the surface of each material. We repeat the same operation for each material in each petri dish. After the operation, we start timing and add 100 ml distilled water to the marked petri dish every 5 min at the time point. Then we recover the leaching solution. After standing for 5 min, we use a spectrophotometer with 540 nm wavelength to measure the optical density values of free hemoglobin in the leaching solution at different time points and then draw the curves of dynamic coagulation.

### Rabbit Ear Artery Hemorrhage Model

We take four white rabbits from New Zealand and use 4% pentobarbital to anesthetize the white rabbits at a dose of 30 mg/kg. We fix their limbs with fixators. We take off their back hair so as to identify their ear artery. After disinfecting the wounds with 75% alcohol, we cut the ear artery by using scalpel and let it bleed for 5 s. After wiping it with gauze, we utilize different materials to stop bleeding of different white rabbits. We press the position for hemostasis treatment. We pressurize the material with 200 g lead sinker and start timing. We remove materials from the bleeding place every 10s and observe whether the bleeding is completely stopped. If the bleeding is not stopped, we continue to press. When the bleeding is completely stopped, we record the hemostatic time using gauze as control. After hemostasis, we weigh the materials and minus its original weigh to calculate the amount of bleeding during the hemostasis process. All experiments are repeated 5 times to avoid computation errors.

### Rat Liver Hemorrhage Model

We take four SD rats and inject 10% chloral hydrate at a dose of 0.4 ml/100 g for anesthesia. We remove their upper abdominal hair, expose the skin, and disinfect it with 75% alcohol. We open the rat’s abdominal cavity layer by layer with a scalpel, and take out the left liver lobe. We cut a 1.5 cm*1.5 cm*0.3 cm wound on its liver lobe. After letting it bleed for 5 s, we wipe the blood in the abdominal cavity with gauze. We quickly place the material on the wound and press it to stop the bleeding, and start timing at the same time. Then. We opened the material for 10 s and observe whether the wound remains bleeding. If it bleeds, we continue to press. If it stops, we record the hemostasis time. After the blood stops exuding, we remove the material from the wound and weigh the material. We subtract the weight of the material that placed and calculate the amount of bleeding. The fish collagen sponge and the control materials are calculated in the same way. All experiments are repeated 5 times to reduce computation errors.

### Whole Blood Reaction

We take 3 ml rat anticoagulated whole blood and add 300 ul 1M CaCl_2_ solution to it. 0.05 g of each materials are placed in a disposable clean petri dish. We drop 1 ml recalcified whole blood to each material, and place the petri dish in a constant temperature incubator of 37°C for 10 min. Then, we take it out and wash it for three times with using 0.2M PBS buffer of pH 7.4. It is then immersed with 2.5% glutaraldehyde solution, which is fixed with 2.5% glutaraldehyde solution for 12 h. We put the fixed materials into a low-temperature freeze dryer. After they are completely dried, we spray gold and observe through a scanning electron microscope. All steps are repeated 5 times.

### Platelet Adhesion

We take 0.01 g for each of the three materials and place them in a disposable clean petri dish. We take three tubes of 2 ml rat anticoagulated whole blood into a centrifuge and prepare platelet-rich plasma (PRP) at 5,000 rpm for 3 min. We take 200ul platelet-rich plasma (PRP) and drop them on the three materials respectively. We place the petri dish in a constant temperature incubator at 37°C to incubate for 10 min. We take out the materials and wash them three times using a 0.2M PBS buffer of pH 7.4, and then soak them with 2.5% glutaraldehyde solution. We fix them with 2.5% glutaraldehyde solution for 12 h. We put the fixed materials into a low-temperature freeze dryer. After they are completely dried, we spray gold to observe via a scanning electron microscope. All steps are repeated 3 times.

### Red Blood Cell Aggregation

We take 6 ml anticoagulated rat whole blood and divide it into three parts evenly, and put each part in a 2 ml centrifuge tube. We discard the upper plasma at 2000rpm for 10 min s. We wash the lower blood cells with normal saline for 5 times to prepare red blood cell suspension with a specific volume of 40%. We place 0.01 g of three materials in a disposable clean petri dish, drop 1 ml red blood cell suspension to each material, and incubate them in a constant temperature incubator at 37°C for 30 min After taking out materials, we continue to wash materials with normal saline for 5 times to remove the free red blood cells and place them at room temperature for 1 h. Finally, the materials are fixed with 2.5% glutaraldehyde solution for 12 h. After gradient elution with 100% ethanol solution, materials are freeze-dried at low temperature. We spray gold to observe via scanning electron microscope and repeat the above steps for three times.

## Results

### Physical and Chemical Characteristics of Collagen and Sponge

**FIGURE 2:**
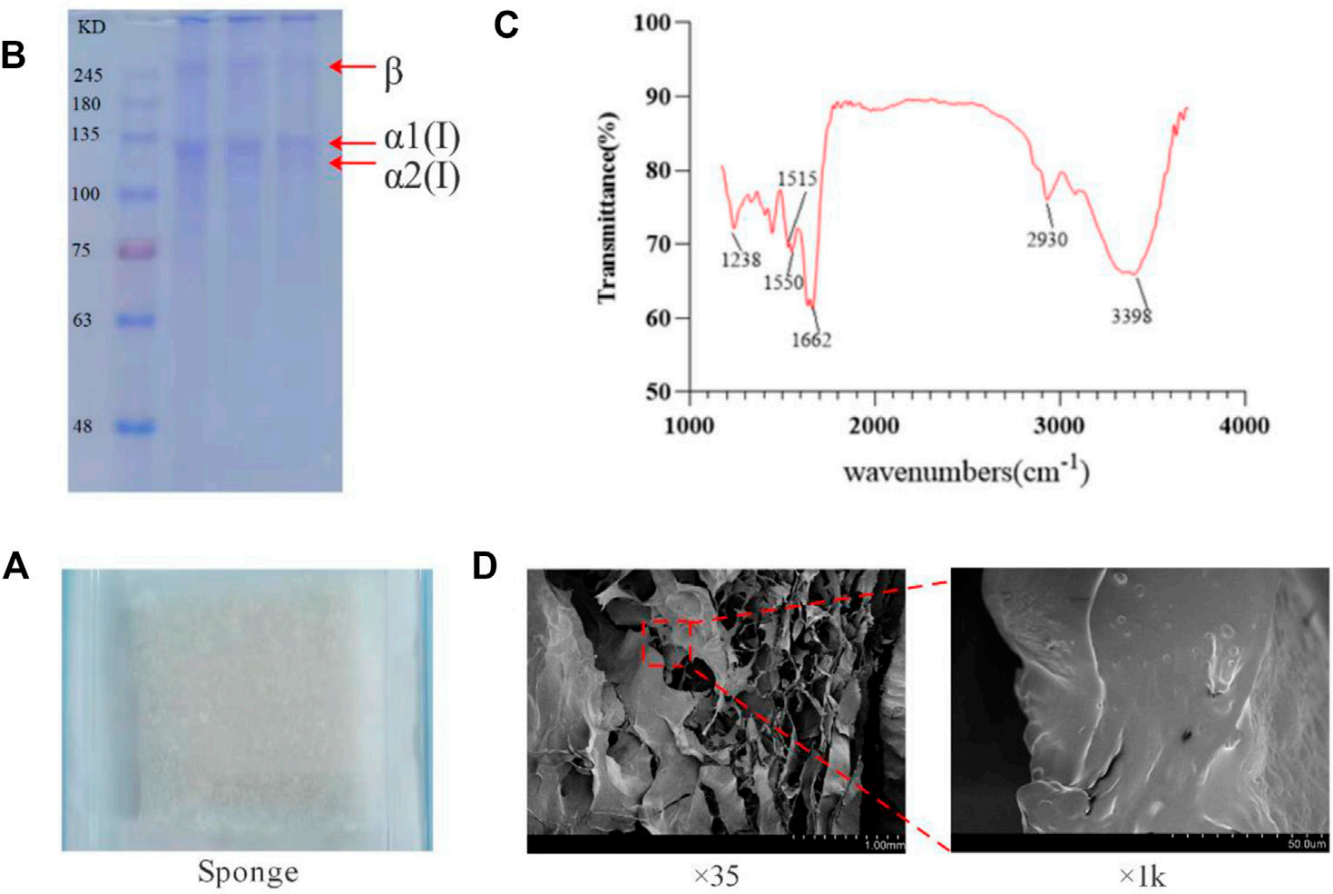
Molecular weight and infrared absorption spectrum of tilapia collogen and sponge microstructure. **(A)**. Irradiation-sterilized collagen sponge. **(B)**. Results of SDS-PAGE electrophoresis of collagen. **(C)**. Infrared absorption spectra of collagen. **(D)**. Electron microscope scan of collagen sponge.

The multi-stage membrane filtration technology utilized in the investigation can effectively remove impurities such as large particles, microorganisms, and small molecules within a short time. It could shorten the production cycle, and the structure of obtained collagen concentrate is in the form of transparent honeycomb (Figure 1), with a high yield of 10%. SDS-PAGE detection results showcase that the protein bands are clear without diffuse bands and tailing, which indicates that the obtained collagen has high purity without degradation or contaminants. The α1, α2 peptide chain bands are clear and complete, and the molecular weights are above 100 KDa. The molecular weight of *β* chain is about 300 KDa and the band is clear. The proportion is high indicating that peptide chains are intact and the original structure of collagen remains intact (Figure 2B). The hydroxyproline content determination results of the samples verify that the average hydroxyproline content is 9.3%, which is in line with the basic characteristics of type I collagen and is agree with the experimental results from literatures.

Natural type I collagen has A specific infrared absorption band, mainly contain amide A band (absorption peak wavelength range 3400–3440 cm^−1^; But if it forms hydrogen bonds with other groups, the absorption summit migrates to the lower frequency region, In the range of 3300–3400 cm^−1^), amide B band (absorption peak wavelength range 2930–2944 cm^−1^), amide I band (absorption peak wavelength range 1625–1690 cm^−1^), and amide II band (absorption peak wavelength range 1510–1550 cm^−1^) and amide III band (absorption peak wavelength range 1235–1245 cm^−1^); They are caused by the stretching vibration of hydrogen bond in N-H and O-H, asymmetric stretching vibration of -CH2, stretching vibration of peptide chain C=O, bending vibration of peptide chain N-H and stretching vibration of C-N respectively. It can show the degree of hydrogen bond bonding within and between collagen peptide chains, and whether the secondary and tertiary structures are intact. As shown in Figure 2C, the extracted collagen showed obvious absorption peaks at 3398 cm^−1^, 2930 cm^−1^, 1662 cm^−1^, 1515 cm^−1^, 1550 cm^−1^, and 1238 cm^−1^, which were in the same position as the absorption peaks of all the infrared absorption bands of natural type I collagen. Therefore, it can be determined that the extracted collagen has all the specific infrared absorption bands of natural type I collagen, and has *β* -folding, triple helix and other advanced structures. It can be determined that the extracted collagen is not damaged, and the peptide chain and triple helix structure are complete and in a natural state.

The freeze-dried tilapia collagen sponge is milky white, porous, fluffy, and tensioned (Figure 2A), and can absorb water rapidly when being placed in pure water. The water absorption rate is 2,100%, which helps the sponge absorb blood from the wound in the hemostasis process. Scanning electron microscopy results show that at low magnification, the sponge is in a regular porous honeycomb shape with a pore having diameter of about 5um and over 85% porosity. At high magnification, the surface of collagen is smooth without any impurities (Figure 2D).

### Destructive Effect on Blood Cells

**FIGURE 3:**
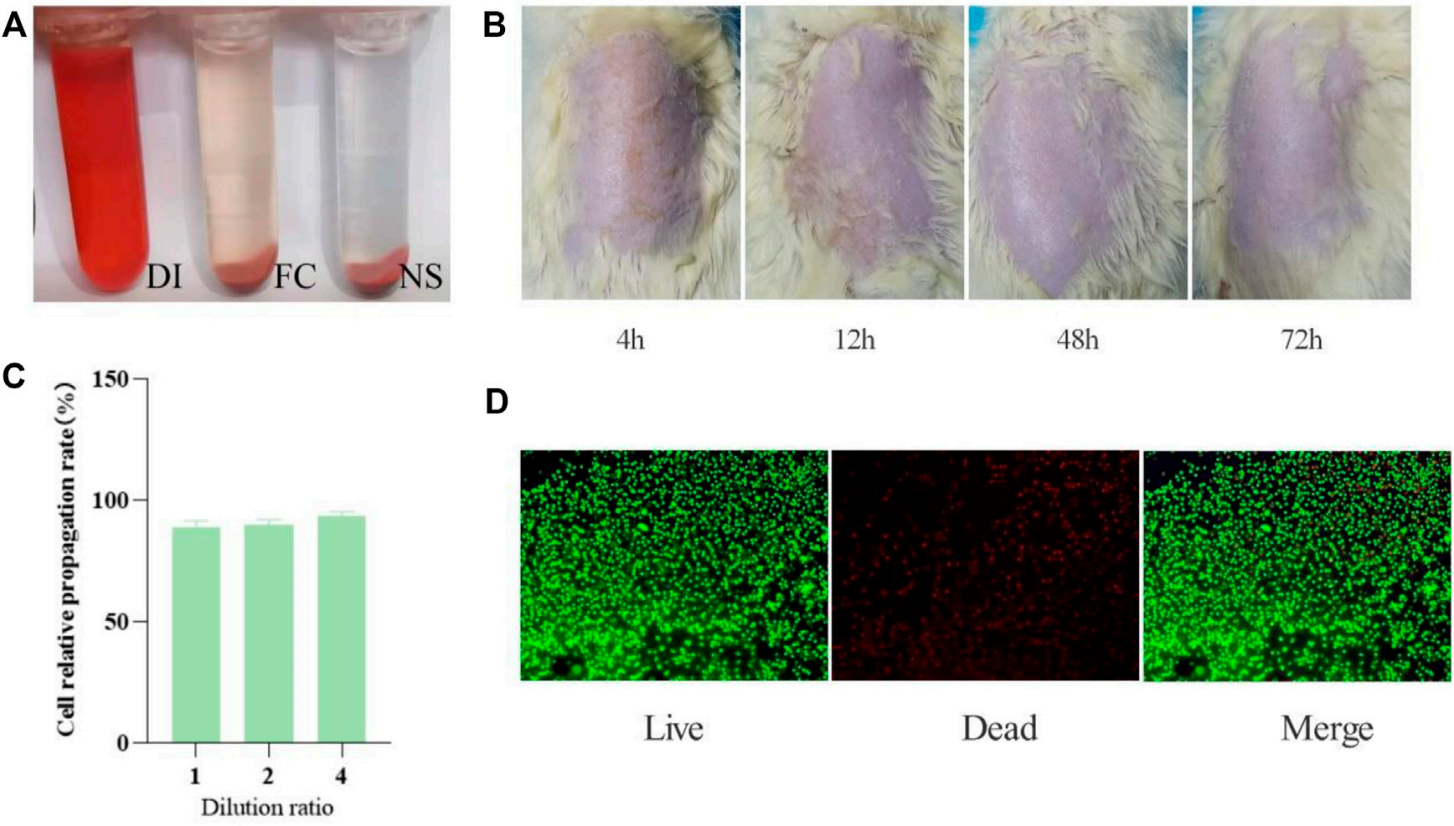
Collagen safety test results. **(A)** Hemolysis test results (*n* = 5 per group). **(B)** Skin irritation results (*n* = 5). **(C)** Cell relative propagation rate (*n* = 3). **(D)** Live-Dead staining of L929 cells seeded on the surface of different composites after culturing for 7 days. Live cells emit green fluorescence, while dead cells emit red fluorescence. Scale bar: 100 μm *n* = 3.

The hemolysis rate is a general standard for evaluating the quality of hemostatic materials. General standards stipulate that if hemolysis rate is less than 5%, the material could meet the clinical blood safety requirements. The fish collagen group (FC) and physiological saline group (NS) are centrifuged. The supernatant of the NS group is light yellow and basically colorless and transparent. The red blood cells are deposited to the bottom of the tube without hemolysis. The FC group is light brown-red, and most of the red blood cells are deposited to the tube bottom, with slight hemolysis. In the positive control group (i.e. distilled water group, DI), the liquid in the tube is red and there is no precipitation at the bottom of the tube. The red blood cells are completely ruptured and dissolved (Figure 3A).

We take the supernatant and measure the absorbance at 545 nm wavelength and repeat this step three times to take the average. Hemolysis rate is defined as (sample absorbance-negative control absorbance)/(positive control absorbance-negative control absorbance) ×100% (Table. 1).

**TABLE 1:** OD and hemolysis rate in each group (*n* = 10, per group).

Groups	OD $(\bar{x}$ ±SD)	Average hemolysis rate (%)
Fish collagen (FC)	0.025 ± 0.003	0.12
Physiological saline (NS)	0.014 ± 0.004	0.01
Distilled water (DI)	1.367 ± 0.004	100

From the above experiment data, the average hemolysis rate of FC group is only 0.12% (less than 5%), which indicates that it will not cause hemolysis.

### Skin Irritation Response

We observe the skin changes of SD rats at 4, 24, 48, and 72 h respectively, and find that the surfaces of every rats’ skin are smoothand without erythema, edema, or scabs, which proves that fish collagen sponge has no skin sensitization (Figure 3B).

### Cell Culture

According to the grading standard of cytotoxicity evaluation (Table. 2), the original solution of fish collagen sponge extract has a cell relative propagation rate of 89% and a cytotoxicity grade of 1. The relative propagation rates of the cells diluted 2 times and 4 times are 91 and 94%, respectively. The cytotoxicity is level 1. According to the standard, when the cytotoxicity is at the first levelit is deemed to be noncytotoxic (Figure 3C). Certainly, the prepared fish collagen sponge is noncytotoxic and will not cause many cell deaths *in vitro*.

**TABLE 2:** Cytotoxicity evaluation grading.

Relative propagation rate (%)	Cytotoxicity grading	Outcome assessment
≥100	0	noncytotoxic
75–99	1	noncytotoxic
50–74	2	Mild cytotoxicity
25–49	3	Moderate cytotoxicity
1–24	4	Moderate cytotoxicity
0	5	Severe cytotoxicity

Cell adhesion can further reflect the compatibility of the material with cells. From the staining results of live/dead cells cultured to 7 days (Figure 3D), the cells have grown to the entire surface of the material and extended to the inside of the material along the scaffold and pores, which fully indicates that the material has good cell compatibility.

### Degradation in the Body

**FIGURE 4:**
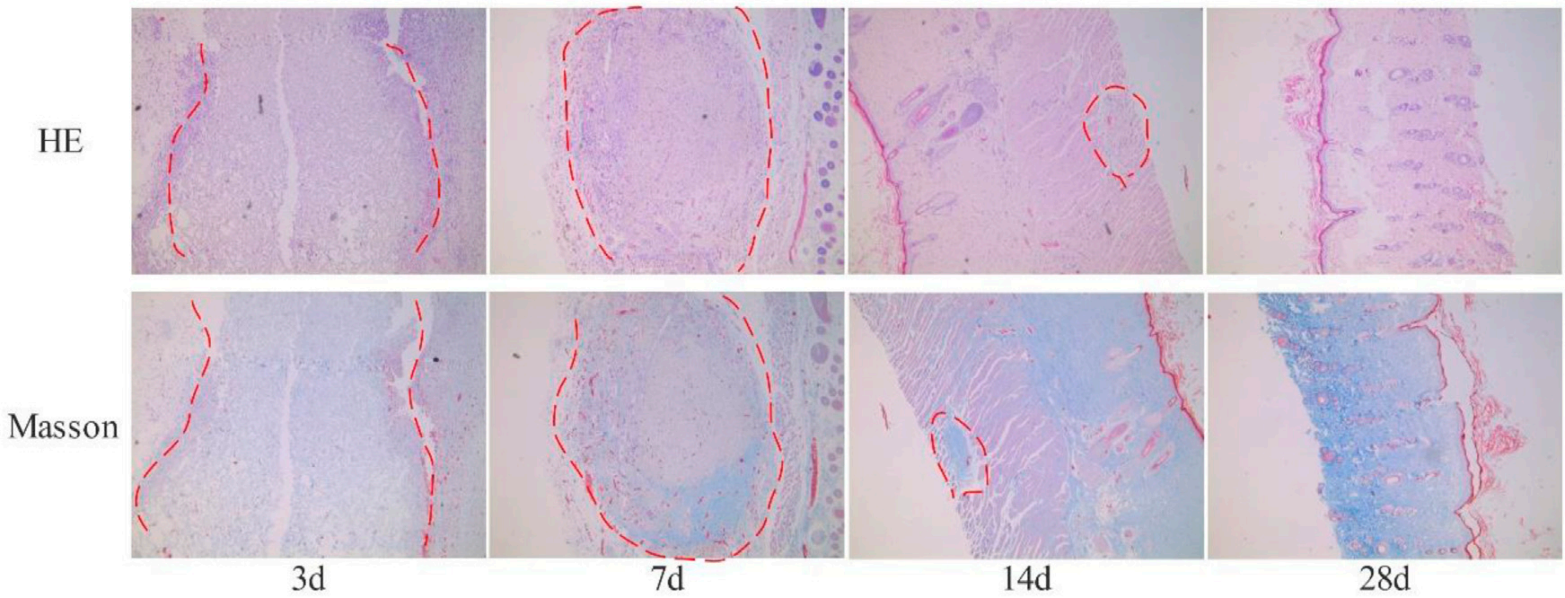
The results of HE and Masson staining (×40) are obtained by subcutaneously embedded fish collagen sponge (The red dotted line shows the implanted fish collagen sponge).

The subcutaneous implantation experiment can effectively evaluate the biocompatibility and degradability of the studied materials. The bovine collagen sponge that is sold one the current market and has passed the safety test can be utilized as a control to accurately evaluate the safety of the fish collagen sponge (Figure 4). On the 3rd day after implantation, the fish collagen sponge is completely wrapped by subcutaneous fibrous tissue, and there are no immune rejections such as redness, swelling and suppuration on the wound surface and subcutaneous tissue. The diameter of the cyst is about 5 mm. On the 7th day, the wrapped material gradually degrades. The diameter of the capsule is about 4 mm. By the 14th day, the diameter of the cyst is about 2 mm. On the 28th day, only tiny cysts with a diameter of less than 1 mm are found under the skin. The material is basically completely degraded, and the degradation rate has reached >90%. The entire degradation process is similar to that of the control bovine collagen sponge. In order to facilitate the observation of the material in the fibrous capsule, sections were made from the skin surface until the fibrous capsule was completely cut, and then the sections were fixed and stained. After staining, clear fibrous structures could be seen in the position of the fibrous capsule wall (the area marked by red dotted line in Figure 4), so that the internal and external structures of the fibrous capsule were completely separated from the material. The results of pathological staining demonstrate that on the 3rd day, the fish collagen sponge is completely wrapped by fibrous tissue and there is a small amount of inflammatory cell infiltration inside the cyst. The sponge is relatively intact. On the 7th day, the inflammatory cells inside the sponge decrease, and both the edges and inside of the sponge begin to degenerate. On the 14th day, only a small number of inflammatory cells are observed inside the sponge. More than half of the sponge are degraded. The staining results on the 28th day show that the cysts are mainly fibrous tissue without any inflammatory cells inside. The sponge is almost completely degraded. Therefore, we could conclude that the fish collagen sponge has good biocompatibility as it will not cause inflammatory reactions or any immune rejections in the body, and it. Meanwhile, it can be completely degraded within the body. Furthermore, the degradation products has excellent degradation properties and will not cause any adverse reactions to the body.

### Blood Absorption Swelling Rate

In the initial stage of hemostasis, the hemostatic material has a fast blood absorption rate on the wound surface. On the one hand, it stops bleeding and other exudates from the wound surface, which plays a physical hemostatic effect while effectively preventing wound infection that caused by fluid accumulation. On the other hand, it can quickly attract platelets and blood cells, interact with them, activate the coagulation pathway, and promote the formation of thrombus on the wound surface. The data show that the fish collagen sponge weight could reach 20 times of the original sponge weight after fully absorbing blood. The blood absorbing rate can reach about 1917 ± 135% (*n* = 10), while the rate of bovine collagen can reach about1632% ± 143% (*n* = 10), and the rate of oxidized regenerated cellulose can reach about 920 ± 127% (*n* = 10). It can be concluded that fish collagen sponge has good water absorption and blood absorption abilities. Therefore, it can be employed to promote wound coagulation through blood concentration at the wound surface and the interaction with platelets as well as blood cells.

### Comparisons Regarding Blood Clotting Index (BCI)

**FIGURE 5:**
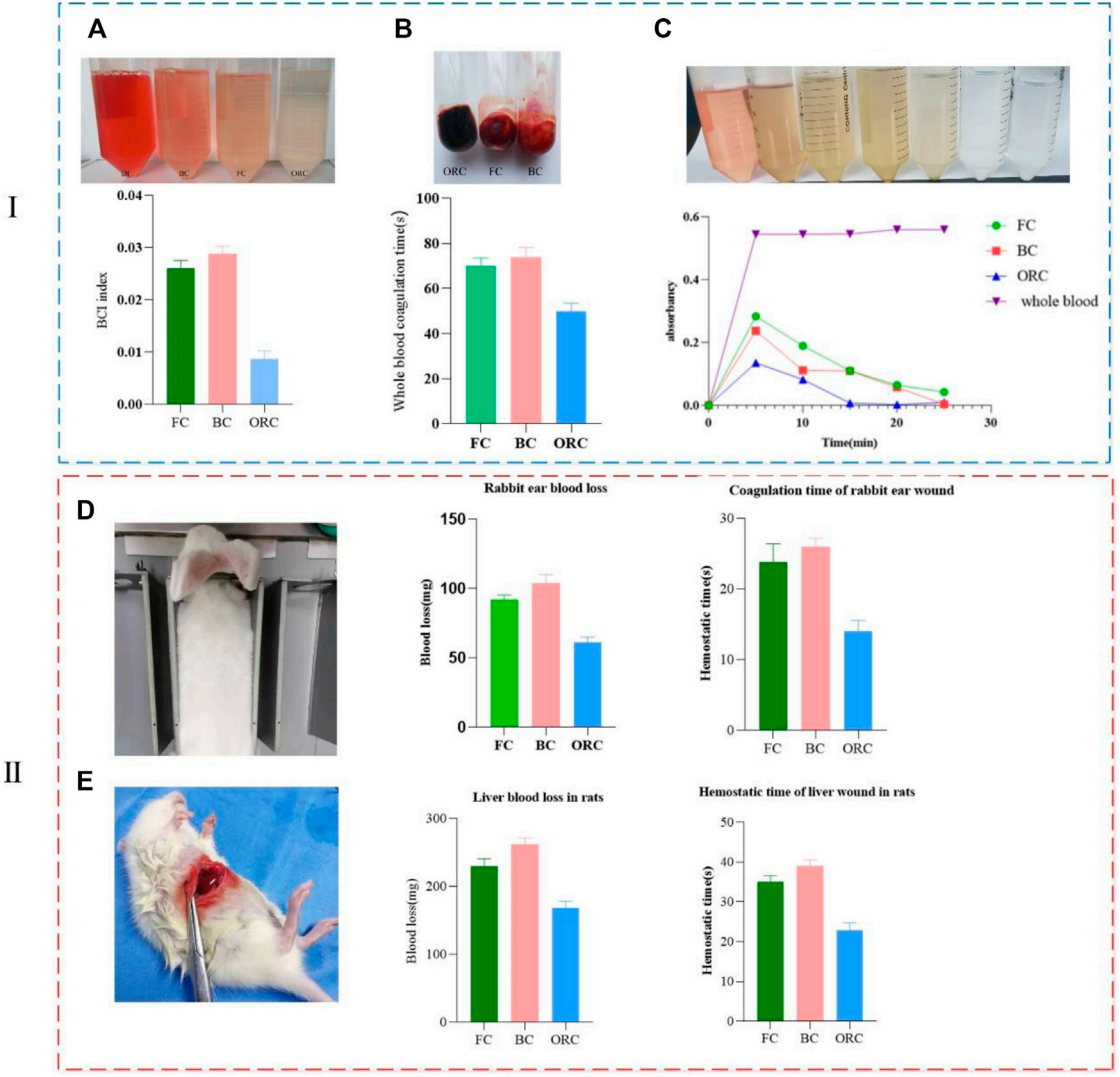
Comparison of clotting ability regarding the three materials *in vitro* (i) and *vivo* (ii) (collagen sponge-FC, bovine collagen sponge-BC, oxidized regenerated cellulose-ORC). **(A)** Comparisons regarding blood clotting index (BCI). **(B)** Whole blood coagulation time. **(C)** Dynamic coagulation curve. **(D)** Coagulation time and blood loss in rabbit ear wound. **(E)** Coagulation time and blood loss in liver wound of rats.

During the hemostasis process, part of the blood could not be coagulated by the hemostatic material. We put it in pure water to obtain free hemoglobin solution. The relative content of free hemoglobin is calculated by measuring OD, which indirectly reflects the effect of the hemostatic material regarding hemoglobin. Adsorption and compatibility with blood reflect the hemostatic ability of the test material. Based on the BCI index determined by the free hemoglobin OD, the hemostatic properties of various materials can be evaluated. The lower the BCI index, the better the clotting effect of the material. After coagulation, we add pure water. Images show that the solution of the reference group with whole blood is red, the solution of the oxidized regenerated cellulose group is almost colorless and transparent, and the solution of the bovine collagen sponge group and the fish collagen sponge group are both light red. The color of the fish collagen sponge group is relatively lighter. The BCI index of fish collagen sponge is 0.026, and the index of bovine collagen sponge is 0.029, which shows the difference between them is very slight. The BCI index of oxidized regenerated cellulose is 0.009, which is significantly different from the two collagen sponges (Figure 5A). This indicates that the adhesion ability of fish collagen sponge to red blood cells and hemoglobin, and the blood coagulation ability are not significantly different from that of bovine collagen sponge. Indices of both collagen sponges are significantly lower than that of oxidized regenerated cellulose.

### Whole Blood Coagulation and Dynamic Clotting Time

After the material is in contact with blood, the coagulation rate and coagulation degree can be detected by visually observing the blood fluidity at different time points as well as measuring the optical density of free hemoglobin. The coagulation rate and coagulation degree are negatively correlated with the optical density. Multiple coagulation experiments with respect to whole blood show that, after dripping blood (Figure 5B), the average coagulation time of fish collagen sponge (FC), bovine collagen sponge (BC), and oxidized regenerated cellulose (ORC) are 70, 75, and 50 s respectively. The average difference in coagulation time between the two collagen sponges is about 5s, which is not significant. The coagulation time differs significantly from that of oxidized regenerated cellulose by 20 and 25 s, respectively. From the absorbance change curve (dynamic coagulation curve) of the three materials at different time points, the absorbance of the fish collagen sponge and bovine collagen sponge effluent was higher than that of the oxidized regenerated cellulose at the 5 min time point. It indicates that within 5 min, the coagulation effect of oxidative regeneration cellulose is higher than those of the other two collagens. At 10 min, the absorbance values of the three materials are significantly reduced, which indicates that the three materials promote blood coagulation, and the coagulation rate of oxidized regenerated cellulose is the highest (Figure 5C). During the whole dynamic coagulation process, the oxidized regenerated cellulose curve has the largest decline and the steepest slope, which infers the best coagulation performance. The curve of fish collagen sponge has the same decline and slope as that of the oxidized regenerated cellulose, yet the value was relatively higher showing that it also has high coagulation performance. Bovine collagen sponge has a relatively small decline, but its value is between that of fish collagen and oxidized regenerated cellulose, which informs its good anticoagulant property.

Combining with blood absorbing rate, BCI, whole blood clotting time and dynamic clotting curve, we find that fish collagen and bovine collagen are much better than oxidized regenerated cellulose in blood absorbing rate, but they are significantly worse than oxidized regenerated cellulose in terms of BCI index and whole blood clotting time. The decline regarding the dynamic coagulation curves of fish collagen and bovine collagen is also lower than that of oxidized regenerated cellulose indicating that fish collagen and bovine collagen mainly function on blood cells and platelets, which fulfill the blood clotting function. The mechanisms of function regarding oxidized regenerated cellulose and the two types of collagen are somewhat different.

### Hemostasis in the Body

In the rabbit ear hemostasis experiment (Figure 5D), we select similar arteries in terms of position and thickness to minimize the error caused by the flow rate and inequal thickness. In the experiment, in addition to the three materials, ordinary medical gauze is employed to stop the bleeding. Since all the other materials are successfully applied to stop bleeding, the wound treated by ordinary medical gauze continues to bleed. According to the statistical analysis regarding the hemostasis time and bleeding volume, the hemostasis time of oxidized regenerated cellulose is 14±1s (*n* = 5), and the bleeding volume is only 60±4 mg (*n* = 5), which has the best hemostatic effect. The hemostasis time of fish collagen sponge is 23±2s (*n* = 5) and the bleeding volume is 90±5 mg (*n* = 5), which make its hemostatic effect in the second place. It is slightly better than bovine collagen sponge (hemostasis time 26±1s (*n* = 5), bleeding volume 100±3 mg (*n* = 5)), but the difference was not significant. During the hemostasis process, we observe that oxidized regenerated cellulose form dark brown plaques on the wound surface in a short time. Fish collagen sponge can quickly absorb blood and form blood crust. Bovine collagen sponge absorbs blood faster than fish collagen. Fish collagen sponge can stop the bleeding in about 26 s. All three materials can adhere to the wound surface and form a certain pressure on it to prevent bleeding.

As one of the most important organs in animals and human body, liver needs to stop bleeding promptly and quickly during clinical surgery, otherwise, it will induce a variety of adverse prognostic reactions, which even make damage to life. In the rat liver hemostasis experiment, we select the same liver lobules in all individuals to make wounds of the same length and width. In this manner, we try to minimize the differences between individuals. The results show that the hemostatic effect of oxidized regenerated cellulose is excellent, and the hemostasis time is only 23±1s (*n* = 5). The bleeding volume is 170±6 mg (*n* = 5), which can quickly form dark brown plaques on the wound and stop wound bleeding. Fish collagen sponge can quickly adhere to the wound surface and form a certain pressure on the wound surface. While physically stopping the bleeding, fish collagen sponge activates the endogenous blood coagulation pathway and hemostasis is achieved within 35±1 s (*n* = 5). The amount of bleeding is 230±5 mg (*n* = 5). The hemostatic process of bovine collagen sponge is similar to that of fish collagen sponge, but the hemostasis time is longer (39±2 s (*n* = 5)) and the bleeding volume is slightly larger (260±3 mg (*n* = 5)). This shows that the hemostatic effect of fish collagen sponge is slightly worse than that of oxidized regenerated cellulose, while slightly better than that of bovine collagen sponge (Figure 5E).

### Adhesion and Aggregation of Blood Cells and Platelets

**FIGURE 6:**
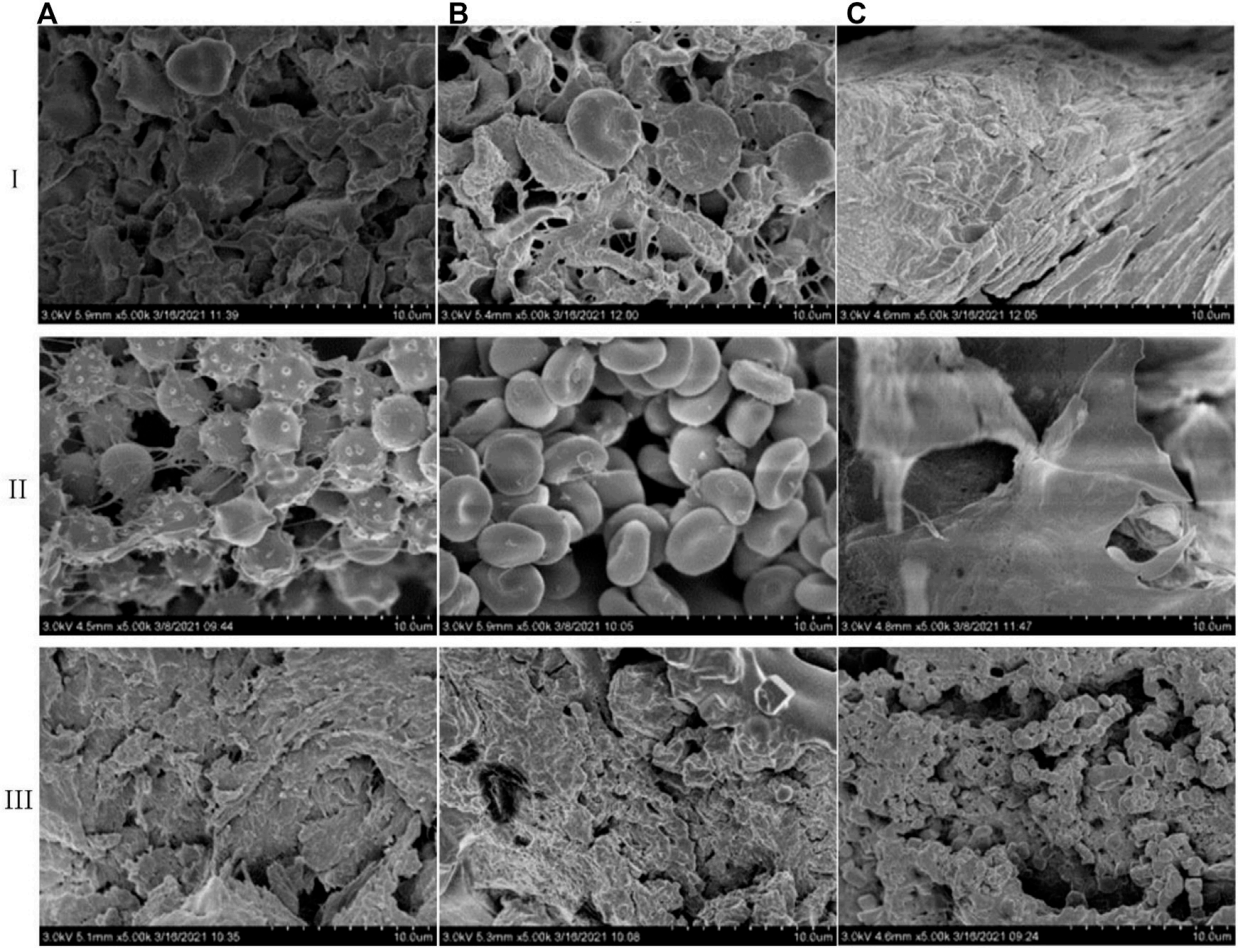
Comparison of action modes of three hemostatic materials on whole blood (i), blood cells (ii), and platelets (iii) (✕5K) **(A)**. Fish collagen **(B)**. Bovine collagen **(C)**. Oxidation regeneration of cellulose).

The surface morphology changes of red blood cells and platelets are the main alternations of the hemostatic material in the hemostasis process. Through the electron microscope scanning of the whole blood after coagulation under three materials (Figure 6i), we find that there are a large number of aggregated plaques on the surface and inside of the pores in the fish collagen sponge (Figure 6Ai). Under high magnification, the platelets are deformed and connected to a whole. Most of the red blood cells have changed in shape and form, which are wrapped by deformed platelets. There are almost no red blood cells with normal shape. Platelets and red blood cells are deformed and highly aggregated to form an intertwined whole. For bovine collagen, there are a large number of plaques on the surface of the protein (Figure Bi). Under high magnification, many deformed platelets wrap many red blood cells and induce deformation. However, some normal red blood cells are still visible, which are also aggregated to form plaques though its density is lower than that of fish collagen. The fibrous structure of oxidized regenerated cellulose is hard to identify and many sheet-like structures are formed on the surface and inside of the structure (Figure 6Ci). Platelets and red blood cells have formed a sheet-like overall structure under high magnification. There is no complete normal structure of platelets and blood cells, both of which form a whole lamella structure.

In order to further validate the effects of the three materials to platelets and blood cells, we add platelets and blood cells to the materials and scan them by electron microscope. The main function of platelets is to accelerate coagulation and hemostasis. Through the rapid response to vascular injury, the coagulation pathway is activated to form thrombus and therefore stop bleeding. This activation process mainly manifests as irregular deformation and pseudopodia protruding and its main functions include activation, adhesion and aggregation. The results of platelet effect experiments show that fish collagen sponge could induce a good number of platelets to adhere and aggregate on the surface as well as the inside of structure (Figure 6Aiii), which deform into dense plaque structures. Bovine collagen sponge mainly enhances platelet aggregation and deformation on the surface of the structure (Figure 6Biii), and the degree of aggregation was slightly lower than that of fish collagen. In the pores of the oxidized regenerated cellulose fiber structure and along the fiber pores (Figure 6Ciii), a good number of platelets aggregate and adhere to each other, which results in deformation. Also, the aggregation degree is similar to that of bovine collagen sponge. The results of red blood cell interaction experiments show that some fish collagen fibers adhere to the surface of red blood cells, which causes many red blood cells to deform and thus form a dense three-dimensional network structure (Figure 6Aii). Red blood cells are stacked on the surface of bovine collagen sponge, and the effect of collagen fibers on red blood cells is unfound (Figure 6Bii). Oxidized regenerated cellulose can induce red blood cells to form a sheet-like structure, and there are no complete normal red blood cells inside the material (Figure 6Cii).

Fish collagen sponge could effectively induce adhesion, deformation and aggregation regarding blood cells and platelets on its surface and inside the cells. Its fibers can bind to certain sites on the surface of platelets or blood cells to increment their aggregation and deformation. Bovine collagen mainly induces platelet adhesion, aggregation, and deformation on the surface, and induces blood cell accumulations on its surface. Oxidized regenerated cellulose can greatly deform platelets and blood cells, which aggregates in large quantities, and forms a sheet-like structure to achieve hemostasis. These findings are consistent with the results from *in vivo* and *in vitro* coagulation performance testing experiments.

## Discussion

Quick hemostasis is the most effective method to increase the survival rate of critically ill patients, and it is also a wound treatment method often used in daily medical treatment and daily life. Various types of hemostatic materials have been widely used in clinical practice due to their excellent hemostatic properties, and collagen which can stop bleeding and repair, has been widely concerned in hemostasis, especially in wound sites that need to be repaired. Collagen has unique triple helix structure and typical (Gly-X-Y) structure (X and Y stand for proline and hydroxyproline, respectively). It has excellent mechanical strength, biocompatibility, biodegradability, and induction of cell proliferation and differentiation, making it excellent in hemostasis. At present, the generally recognized mechanisms of collagen hemostasis are: 1) Collagen molecules can continuously absorb blood after contact with the wound surface by virtue of their excellent hydrophilic properties, adhere to the surface of the wound surface, form blood crusts, and block the rupture of blood vessels. 2) It can induce platelet aggregation, stimulate platelets to release coagulation factors, and activate the endogenous coagulation pathway. The two mechanisms cooperate with each other to achieve efficient hemostasis. Additionally, because of its biodegradability, collagen can be de-graded by itself at the wound surface, so there is no need to take it out again, which reduces the occurrence of secondary trauma. Therefore, collagen with a controllable source and good quality is an important raw material for clinically such products. In our study, we use tilapia fish skin that is left from the former procedure. After a series of separation and purification, impurities and microorganisms were removed while obtaining high-purity macromolecular collagen, which effectively improved the utilization efficiency of fish skin and reduced solid waste. At the same time, it offers a recommendation to the application of fish collagen in hemostatic materials.

### Fish Collagen Extraction, Physical and Chemical Properties, and Biological Safety Testing

The entire extraction process was carried out in an environment of 4°C, which can effectively protect the structure of fish collagen and peptide chains from being damaged. The crude extract used a gentle multi-stage membrane filtration system to reduce ex-traction time and loss while effectively removing impurities, microorganisms, mall peptides, inorganic salts, and other ingredients to finally prepare high-purity fish collagen. SDS-PAGE electrophoresis showed that the molecular weights of the α1 and α2 peptide chains were both greater than 120kDa, and the *β* chain was around 300 kDa, with clear bands and without any contaminated band, indicating the high purity of the extracted collagen. The 9.3% hydroxyproline content and the characteristic absorption peaks of amide I, II, and III shown by the infrared absorption spectrum fully indicate that the extracted fish collagen is type I collagen, which is the same as the main type of fish skin collagen shown in the literature. The level of water absorption reflects the hydrophilic properties of the hemostatic material, and determines the degree of hemostatic material sucking blood at the wound surface. The research results show that fish collagen has a similar blood absorption rate to the bovine collagen used in clinical applications, which preliminarily prove that the hemostatic material has a similar blood absorption rate.

Important means including hemolysis test, skin sensitization test, subcutaneous implantation test, cytotoxicitycan test whether implanted medical materials and hemostatic materials are qualified. They are compared with products such as regenerated oxidized cellulose and bovine collagen that have been used in clinical practice for many years. It was found that no adverse reactions occurred, and fish collagen could be gradually degraded in the body, indicating tits good biosafety and biodegradability.

### *In vivo* and *in vitro* Coagulation Performance Testing

The study mainly used blood-sucking rate, BCI index, whole blood coagulation time and animal wound hemostasis to verify the coagulation performance of fish collagen *in vivo* and *in vitro*. Among them, the blood-sucking rate mainly reflects the extent of the material sucking blood on the wound surface, and then the ability of the material to physically stop bleeding in the early stage; the coagulation time of whole blood can reflect the speed of the material to stop bleeding; the BCI index and the dynamic coagulation index can be measured by the free hemoglobin concentration, reflecting the degree of coagulation of the material to the blood; hemostasis on animal wounds is the most direct reflection of the hemostatic properties of hemostatic materials. Studies have found that fish collagen is not much different from commercially available bovine collagen hemostatic sponges in terms of coagulation rate, coagulation speed, coagulation degree, and coagulation in the body, but they are both weaker than regenerated oxidized cellulose, indicating that the coagulation performance of fish collagen is better and has great application value in the market.

### Study on the Mechanism of Blood Coagulation

The coagulation pathway is divided into endogenous coagulation and exogenous coagulation. All hemostatic materials mainly work from one aspect or two at the same time. Meanwhile, some hemostatic materials also have a certain physical hemostatic effect. The coagulation mechanism of terrestrial animal collagen has been explored and studied, mainly sucking blood to block blood exudation, interacting with platelets, and activating the endogenous coagulation pathway. This research conducts a preliminary exploration of the coagulation mechanism of fish collagen and it studies its effect on blood cells, platelets, and whole blood. The results show that fish collagen can bind to specific sites on the surface of blood cells and platelets when acting with a single component, and induce the aggregation of blood cells and platelets, and the whole blood reaction can induce blood agglutination; it can be judged that fish collagen can interact with blood, induce it to accumulate on the wound surface, form a blood clot, squeeze the wound surface, and block the continuous blood oozing from the blood vessel; it can be inferred when it interacts with platelets, and it may also induce endogenous coagulation mechanisms.

The research explored the process of extracting collagen from fish skin, and apply a method (i.e. by using a multi-stage membrane filtration device) to obtain high-purity collagen in one step, and tested its physical and chemical properties, biological safety, and biodegradability; And its coagulation performance and mechanism have been preliminarily explored, but further research on coagulation path is needed.

## Conclusion

The fish collagen extracted from tilapia skin has typical characteristics of type I collagen with complete high-level structures such as *β*-sheets and triple helices, and the natural state is maintained in good condition. The prepared collagen sponge has a good 3D structure, good porosity, water absorption and mechanical properties. Also, it has good biocompatibility, which meets the requirements for absorbable hemostatic materials. Hemostatic performance testing results illustrate that fish collagen exhibits excellent hemostatic properties *in vivo* and *in vitro*, which can achieve hemostasis within a short period of time to meet the clinical requirements for hemostatic materials. We conclude that certain sites on the fish collagen peptide chain could bind to specific sites on blood cells and platelets, which thereby activate the coagulation pathway and achieve the goal of coagulation within a short time period. The specific molecular mechanism calls for further investigations.

## Data Availability

The original contributions presented in the study are included in the article/Supplementary Materials, further inquiries can be directed to the corresponding author.
